# Impact of clinical-pathological factors on locoregional recurrence in mastectomy patients with T1-2N1 breast cancer: who can omit adjuvant radiotherapy?

**DOI:** 10.1007/s10549-021-06378-2

**Published:** 2021-09-06

**Authors:** Xiaofang Wang, Li Zhang, Xiaomeng Zhang, Jurui Luo, Xuanyi Wang, Xingxing Chen, Zhaozhi Yang, Xin Mei, Xiaoli Yu, Zhen Zhang, Xiaomao Guo, Zhimin Shao, Jinli Ma

**Affiliations:** 1 Department of Radiation Oncology, Fudan University Shanghai Cancer Center, Shanghai, 200032 China; 2 Department of Breast Surgery, Fudan University Shanghai Cancer Center, Shanghai, 200032 China; 3 Department of Oncology, Shanghai Medical College, Fudan University, Shanghai, 200032 China; 4 Shanghai Key Laboratory of Radiation Oncology, Shanghai, 200032 China

**Keywords:** Breast cancer, Early stage, PMRT, Prognostic factors

## Abstract

**Purpose:**

Postmastectomy radiation therapy (PMRT) in T1–T2 tumors with 1–3 positive axillary lymph nodes (ALNs) is controversial. This study was to identify prognostic factors of locoregional control (LRC) following mastectomy with or without PMRT for patients with T1-2N1 breast cancer and to discuss the selection of patients who might omit PMRT.

**Materials and methods:**

Between January 2006 and December 2012, the data of 1474 postmastectomy patients staged pT1-2N1 were analyzed. PMRT was applied in 663 patients. LRC and disease-free survival (DFS) were calculated using the Kaplan–Meier method. Cox regression model was applied in the univariate and multivariate analyses to recognize the recurrence risk factors.

**Results:**

With the median follow-up duration of 93 months (range, 5–168 months), 78 patients (5.3%) failed to secure LRC and 220 patients (14.9%) experienced any recurrence. The 7.7-year LRC and DFS was 94.9% and 85.4% respectively in the entire cohort. PMRT significantly improved 7.7-year LRC from 93.4% to 96.6% (*p* = 0.005), but not the DFS (*p* = 0.335). Multivariate analysis revealed that PMRT was an independent prognostic factor of LRC (*p* < 0.001), meanwhile, age ≤ 40 years (*p* = 0.012), histological grade 3 (*p* = 0.004), 2–3 positive nodes (*p* < 0.001) and tumor size of 3–5 cm (*p* = 0.045) were significantly associated with decreased LRC. The 7.7-year LRC for patients with 0, 1, and 2–4 risk factors was 97.7% / 98.9% (*p* = 0.233), 95.3% / 98.0% (*p* = 0.092), and 80.3% / 94.8% (*p* < 0.001) in the non-PMRT and PMRT group, respectively.

**Conclusions:**

In patients with T1-2N1 breast cancer, clinical-pathological factors including young age, histological grade 3, 2–3 positive nodes, and tumor size of 3–5 cm were identified to be predictors of a poorer LRC following mastectomy. Patients with 0–1 risk factor might consider the omission of PMRT.

## Introduction

Postmastectomy radiotherapy (PMRT) has long been the standard for patients with tumors larger than 5 cm or with 4 or more positive axillary lymph nodes (ALNs). However, for early staged patients with T1–2 tumors and 1–3 positive ALNs, the role of PMRT remains controversial. The use of PMRT is supported by the 15-year result of EORTC 22922/10925 [1] and the 20-year result of the Early Breast Cancer Trialists’ Collaborative Group (EBCTCG) meta-analysis [2], which showed significant reductions of locoregional recurrence (LRR) and breast cancer mortality by PMRT. Based on these, the NCCN guideline strongly recommends the locoregional irradiation in patients staged pT1-2N1 in recent years [3]. However, some concerns were evoked from today’s perspective: patients who participated were not all staged at pT1-2N1; novel systemic treatment was not available at the time of some trials conducted; in terms of toxicity, the application of novel radiation techniques is expected to further reduce radiation-associated heart disease. So as proposed by ASTRO and St. Gallen Consensus [4, 5], PMRT should be conducted individually in consideration of risk factors and toxicity in the early staged patients.

Previous studies have recognized several risk factors, such as patient age, tumor size, number and ratio of positive lymph nodes, molecular subtype, and lymph-vascular invasion (LVI) [6–9]. However, how to stratify patients into different risk groups was not well defined. The purpose of this retrospective study was to make PMRT decision recommendations according to risk stratification in the era of modern medicine.

## Methods and materials

### Patients

Of all breast cancer patients diagnosed at Fudan University Shanghai Cancer Center between January 2006 and December 2012, 1621 female patients underwent mastectomy and were staged pT1-2N1. The medical records were extracted from the computerized database. Patients who had contralateral advanced breast cancer (*n* = 9), or with neoadjuvant systemic therapy (*n* = 57), or with follow-up time shorter than 3 months (*n* = 81) were excluded. The final cohort included 1474 patients for retrospective analysis. The review of data was approved by the Ethical Committee and Institutional Review Board of our center.

The clinical-pathological information of eligible patients was collected, including age at diagnosis, laterality, tumor location, tumor histology, histological grade, tumor size, number of positive and examined lymph nodes, LVI, and estrogen receptor (ER), progesterone receptor (PR), human epidermal receptor 2 (HER2) status. ER and PR status were evaluated by immunohistochemistry (IHC). A cutoff value of 1% was used to dichotomize cases into positive and negative [10]. Hormonal receptor (HR) + was defined as ER + and/or PR + , and HR- as both ER- and PR-. HER2 status was determined by IHC as well. Tumors were considered HER2 positive if they scored 3 + , indeterminate if 2 + , and negative if 1 + or 0 on IHC. When IHC was indeterminate, tumors were considered HER2 positive with amplification (ratio >  = 2.0) by fluorescence in situ hybridization (FISH) analysis [11].

### Treatment

All patients underwent mastectomy with negative surgical margins and axillary dissection. Following surgery, adjuvant chemotherapy was given according to international guidelines. HR-positive patients received adjuvant endocrine therapy. HER2-positive patients were given anti-HER2 therapy.

Following chemotherapy, PMRT was administered at the discretion of treating physician. Generally, a dose of 50 Gy in 25 fractions was delivered to the ipsilateral chest wall (CW) for all patients, and the regional nodes for node-positive patients, which included supraclavicular (SCV) and infraclavicular (ICV) with or without internal mammary nodes (IMN), using 3D forward field-in-field planning or simplified inverse-planning intensity-modulated RT technique.

### Endpoints

Follow-up data was last updated on Oct 31, 2020. The primary endpoint of this study was locoregional control (LRC), defined as clinical, radiographic, or pathological evidence of LRR within ipsilateral CW and/or regional nodes (i.e. ipsilateral ALN, SCV, ICV, or IMN). Recurrences at other sites except for local–regional were considered distant metastases (DM). The secondary endpoint was disease-free survival (DFS), measured from the date of surgery to the time of first LRR, DM, death, or the last visit.

### Statistical analysis

Patient characteristics between PMRT and non-PMRT subgroups were compared using the Pearson’s *χ*2. The probabilities of LRC and DFS were calculated using the Kaplan–Meier method and compared between groups using the log-rank test. Recurrence risk factors were recognized using Cox regression model in the univariate and multivariate analyses, and subsequently used to stratify patients into low-, intermediate-, and high-risk groups. Sensitivity analysis using the EM algorithm was conducted to verify the stability of the results. Competing-risk analysis was performed to evaluate LRC. The level of significance was set at *p* < 0.05 (two-sided), using SPSS 26.0.

## Results

### Patient characteristics

Among the 1474 patients analyzed, the median age was 51 years (range, 23–86 years). 1418 patients (96.2%) were diagnosed with invasive ductal carcinoma. The median invasive tumor size was 2.3 cm (range, 0.1–5 cm). The primary tumor staging was T1 in 45.3% of patients (*n* = 668), and T2 in 54.7% of patients (*n* = 806). The median number of ALNs examined was 17 (range, 10–39); the percentage of patients having 1, 2, and 3 positive lymph nodes was 52.2% (*n* = 770), 28.9% (*n* = 426), and 18.9% (*n* = 278), respectively. On IHC staining, HR was positive in 79.5% of patients (*n* = 1172). HER2 was positive in 20.6% of patients (*n* = 304).

Adjuvant chemotherapy was administered in 93.1% of patients (*n* = 1373). The most common regimen was the combination of anthracycline and taxane (935; 68.1%), followed by anthracycline-based or taxane-based regimens. About 97.8% of patients completed 4–8 cycles of adjuvant chemotherapy before radiotherapy (RT). Adjuvant endocrine therapy was administered in 95.1% (*n* = 1115) of HR-positive patients, and anti-HER2 therapy was in 47.4% (*n* = 144) of HER2-positive patients. The most common anti-HER2 regimen was one-year trastuzumab alone (125; 86.8%), followed by one-year trastuzumab plus lapatinib, a small-molecular tyrosine inhibitor (15; 10.4%).

PMRT was applied in 45.0% of patients (*n* = 663). Of them, 1 patient received irradiation to CW alone; 592 patients received irradiation to both CW and regional lymph nodes, including 417 patients (70.4%) to CW + SCV/ICV and 175 patients (29.6%) to CW + SCV/ICV + IMN. Of the remaining 70 patients who received RT, the specific treatment field details were not available. A prescription dose of 50 Gy (range, 44-52 Gy) in 25 fractions (range, 22–26) was delivered with no additional boost to local–regional site. 3D forward field-in-field planning was applied in 90% of patients, and simplified inverse-planning intensity-modulated RT technique in 10% of patients.

Table 1 compared patients’ clinical and treatment characteristics between PMRT and non-PMRT subgroups. Patients associated with high risk factors were more likely to be directed to PMRT, including young age, high histological grade, LVI + , larger tumor, advanced lymph nodes, and unfavorable molecular subtypes.

**Table 1:** Patient characteristics and comparison between PMRT and non-PMRT subgroups

Parameters		Total	Non-PMRT	PMRT	*P*
		No. (%)	No. (%)	No. (%)	
Age	≤ 40	237 (16.1)	98 (12.1)	139 (21.0)	< .001
	> 40	1237 (83.9)	713 (87.9)	524 (79.0)	
Laterality	Left	758 (51.4)	415 (51.2)	343 (51.7)	.830
	Right	716 (48.6)	396 (48.8)	320 (48.3)	
Tumor location	Medial	347 (23.5)	167 (20.6)	180 (27.1)	.003
	Central	96 (6.5)	50 (6.2)	46 (6.9)	
	Outer	1028 (69.7)	594 (73.2)	434 (65.5)	
	Unknown	3 (0.2)	0 (0.0)	3 (0.5)	
Histological grade	Grade 1	16 (1.1)	9 (1.2)	7 (1.1)	.004
	Grade 2	926 (62.8)	538 (66.3)	388 (58.5)	
	Grade 3	456 (30.9)	219 (27.0)	237 (35.7)	
	Unknown	76 (5.2)	45 (5.5)	31 (4.7)	
Tumor size	≤ 3 cm	1212 (82.2)	697 (85.9)	515 (77.7)	< .001
	3-5 cm	262 (17.8)	114 (14.1)	148 (22.3)	
No. of positive nodes	1	770 (52.2)	528 (65.1)	242 (36.5)	< .001
	2	426 (28.9)	193 (23.8)	233 (35.1)	
	3	278 (18.9)	90 (11.1)	188 (28.4)	
Lymph-vascular invasion	Negative	510 (34.6)	348 (42.9)	162 (24.4)	< .001
	Positive	825 (56.0)	354 (43.6)	471 (71.0)	
	Unknown	139 (9.4)	109 (13.4)	31 (4.5)	
Biologic subtype	HR + /HER2-	914 (62.0)	529 (65.2)	385 (58.1)	< .001
	HR + /HER2 +	182 (12.3)	76 (9.4)	106 (16.0)	
	HR-/HER2 +	122 (8.3)	54 (6.7)	68 (10.3)	
	HR-/HER2-	157 (10.7)	82 (10.1)	76 (11.3)	
	HR + /HER2 unknown	76 (5.2)	53 (6.5)	23 (3.5)	
	HR− /HER2 unknown	24 (1.6)	17 (2.1)	6 (0.9)	
Adjuvant chemotherapy	No	101 (6.9)	93 (11.5)	8 (1.2)	< .001
	Yes	1373 (93.1)	718 (88.5)	655 (98.8)	
Chemotherapy regimen	Anthracycline	246 (17.9)	184 (25.6)	62 (9.5)	
	Taxane	166 (12.1)	89 (12.4)	77 (11.8)	
	Anthracycline + Taxane	935 (68.1)	423 (58.9)	512 (78.2)	< .001
	Other	15 (1.1)	12 (1.7)	3 (0.5)	
	Unknown	11 (0.8)	10 (1.4)	1 (0.2)	
Hormonal therapy	No	325 (22.0)	165 (20.3)	160 (24.1)	.001
	Yes	1134 (76.9)	632 (77.9)	502 (75.7)	
	Unknown	15 (1.0)	14 (1.7)	1 (0.2)	
Hormonal therapy (in HR + patients)	No	42 (3.6)	23 (3.5)	19 (3.7)	.018
	Yes	1115 (95.1)	621 (94.4)	494 (96.1)	
	Unknown	15 (1.3)	14 (2.1)	1 (0.2)	
Anti-Her2 therapy	No	1327 (90.0)	759 (93.6)	568 (85.7)	< .001
	Yes	147 (10.0)	52 (6.4)	95 (14.3)	
Anti-Her2 therapy (in Her2 + patients)	No	160 (52.6)	80 (61.5)	80 (46.0)	.007
	Yes	144 (47.4)	50 (38.5)	94 (54.0)	

*PMRT* postmastectomy radiotherapy, *HR* hormonal receptor

### Recurrence and survival outcomes

**Fig. 1:**
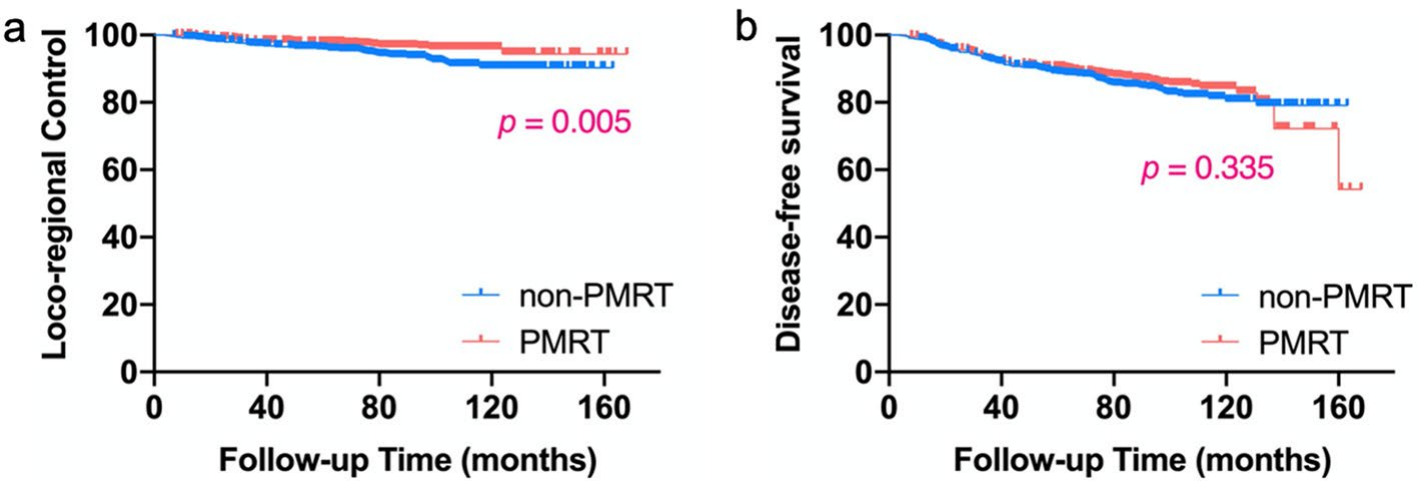
Locoregional control (**a**) and disease-free survival (**b**) of patients with or without PMRT for the entire cohort. *PMRT* postmastectomy radiotherapy

With the median follow-up duration of 93 months (range, 5–168 months), a total of 78 patients (5.3%) developed LRR. Overall, the most common recurrence site was regional nodes alone (55.1%), followed by CW alone (32.1%). Of regional recurrences, the most common site was SCV/ICV, followed by IMN. Table 2 compared the anatomical distribution of LRR between PMRT and non-PMRT subgroups, but the Chi-square test did not show a statistically significant difference (*χ*^2^ = 2.54, *p* = 0.281). For the entire cohort, the 7.7-year cumulative LRC was 94.9%. PMRT significantly improved 7.7-year LRC from 93.4% to 96.6% (*p* = 0.005) (Fig. 1a).

**Table 2:** Patterns of locoregional recurrence

Parameters	Total (*n* = 78)	Non-PMRT (*n* = 54)	PMRT (*n* = 24)
	No. (%)	No. (%)	No. (%)
CW alone	25 (32.1)	15 (27.8)	10 (41.7)
Regional nodes alone	43 (55.1)	33 (61.1)	10 (41.7)
Axilla alone	2	2	0
IMN alone	5	2	3
SCV/ICV alone	31	25	6
Multiple regions	5	4	1
CW + regional nodes	10 (12.8)	6 (11.1)	4 (16.7)

*PMRT* postmastectomy radiotherapy, *CW* chest wall, *IMN* internal mammary nodes, *SCV* supraclavicular, *ICV* infraclavicular

By the date of the last follow-up, 220 (14.9%) patients experienced any recurrence. Among these, 209 patients had DM, including 67 patients with concomitant LRR; and 11 patients had isolated LRR. Table 3 compared the recurrence patterns between PMRT and non-PMRT subgroups, and the difference was statistically significant (χ^2^ = 7.652, *p* = 0.022). For the entire cohort, the actuarial 7.7-year DFS was 85.4%. No statistically significant difference was observed between the non-PMRT and PMRT subgroups for the 7.7-year DFS interval (84.2% vs 86.7%, *p* = 0.335) (Fig. 1b).

**Table 3:** Patterns of recurrence at any site

Parameters	Total (*n* = 220)	Non-PMRT (*n* = 125)	PMRT (*n* = 95)
	No. (%)	No. (%)	No. (%)
LRR alone	11 (5.0)	8 (6.4)	3 (3.2)
DM alone	142 (64.5)	71 (56.8)	71 (74.7)
LRR + DM	67 (30.5)	46 (36.8)	21 (22.1)

*PMRT* postmastectomy radiotherapy, *LRR* locoregional recurrence, *DM* distant metastasis

### Prognostic risk factors

The correlation of LRC and DFS with various prognostic factors is shown in Table 4. In univariate analysis, multiple factors including age, histological grade, number of positive ALNs, and PMRT were significantly associated with LRC. With the exception of PMRT, these factors along with tumor size were also significantly associated with DFS. Triple-negative breast cancer was associated with worse LRC and DFS in comparison with other molecular subtypes, however, the difference was not statistically significant. Additionally, subgroup analysis showed that the application of anti-HER2 therapy could improve both LRC (*p* = 0.022) and DFS (*p* < 0.001) in HER2-positive patients, and the use of endocrine therapy could improve DFS (*p* = 0.015) in HR-positive population.

**Table 4:** Univariate analyses of patient clinical and treatment-related factors for LRC and DFS

Parameters	7.7-year LRC (%)	*P*	7.7-year DFS (%)	*P*
Age		**.032**		**.015**
≤ 40	92.2		80.8	
> 40	95.4		86.2	
Laterality		.633		.605
Left	94.5		85.1	
Right	95.3		85.7	
Tumor location		.898		.392
Medial	94.8		87.5	
Central	94.8		84.9	
Outer	95.3		83.1	
Histological grade		**.019**		**.004**
Grade 1	100.0		93.8	
Grade 2	96.0		87.0	
Grade 3	92.1		80.7	
Grade 1 versus Grade 2		.333		.308
Grade 2 versus Grade 3		.009		.002
Tumor size		.070		** < .001**
≤ 3	95.5		87.3	
3–5	91.9		76.2	
No. of positive nodes		**.018**		**.002**
1	96.6		88.5	
2	93.5		81.6	
3	92.2		82.5	
1 versus 2		.022		.001
2 versus 3		.717		.617
Lymph-vascular invasion		.719		.286
Negative	94.3		87.0	
Positive	94.9		84.4	
Biologic subtype		.200		.400
HR + /HER2−	95.2		86.5	
HR + /HER2 +	95.8		83.8	
HR− /HER2 +	95.3		86.7	
HR− /HER2-	91.2		81.2	
Adjuvant chemotherapy		.377		.254
No	97.7		89.3	
Yes	94.7		85.1	
PMRT		**.005**		.335
No	93.4		84.2	
Yes	96.6		86.7	
Hormonal therapy (in HR + patients)		.121		**.015**
No	92.0		74.3	
Yes	95.4		86.1	
Anti-HER2 therapy (in HER2 + patients)		**.022**		** < .001**
No	93.6		78.3	
Yes	98.4		92.5	

All statistical tests were two-sided and a *p* value of < 0.05 was considered statistically significant (presented as bold)

*LRC* locoregional control, *DFS* disease-free survival, *PMRT* postmastectomy radiotherapy, *HR* hormonal receptor

Advanced multivariate analysis confirmed that clinical-pathological factors including younger age of ≤ 40 years (adjusted hazard ratio [HR], 2.02; 95% confidence interval [CI], 1.17–3.50; *p* = 0.012), tumor size of 3–5 cm (HR, 1.73; 95% CI, 1.01–2.97; *p* = 0.045), histological grade 3 (HR, 1.97; 95% CI, 1.24–3.12; *p* = 0.004), 2–3 positive ALNs (HR, 2.46; 95% CI, 1.51–3.99; *p* < 0.001), and no PMRT delivery (HR, 3.36; 95% CI, 2.11–6.14; *p* < 0.001) were significantly associated with a poorer LRC. Besides the newly identified factor of PMRT (HR, 1.63; 95% CI, 1.22–2.18; *p* = 0.001), younger age of ≤ 40 years, tumor size of 3-5 cm, histological grade 3, and 2–3 positive ALNs remained independent predictors of a shorter DFS interval (Table 5). Therefore, these four clinical-pathological risk factors including young age, tumor size of 3-5 cm, 2–3 positive ALNs, and high histological grade were involved in the following risk group analysis.

**Table 5:** Multivariate analysis of prognostic factors for outcomes of LRC and DFS

Parameters	LRC		DFS	
	HR (95% CI)	*p*-value	HR (95% CI)	*p*-value
Age (years) (≤ 40 vs > 40)	2.02 (1.17–3.50)	.012	1.57 (1.12–2.20)	.008
Histological grade (grade 3 vs grade 1 ~ 2)	1.97 (1.24–3.12)	.004	1.55 (1.17–2.04)	.002
No. of positive nodes (2 ~ 3 vs 1)	2.46 (1.51–3.99)	< .001	1.69 (1.27–2.24)	< .001
Tumor size (3-5 cm vs 0-3 cm)	1.73 (1.01–2.97)	.045	1.91 (1.40–2.59)	< .001
PMRT (no vs yes)	3.36 (2.11–6.14)	< .001	1.63 (1.22–2.18)	.001

*LRC* locoregional control, *DFS* disease-free survival, *CI* confidence interval, *HR* hazard ratio, *PMRT* postmastectomy radiotherapy

### Outcomes of risk groups

In total, 1398 patients were stratified into three groups by recurrence risk, including 377 patients (27.0%) in low-risk group (0 risk factor), 572 patients (40.9%) in intermediate-risk group (1 risk factor), and 449 patients (32.1%) in high-risk group (2–4 risk factors).

**Fig. 2:**
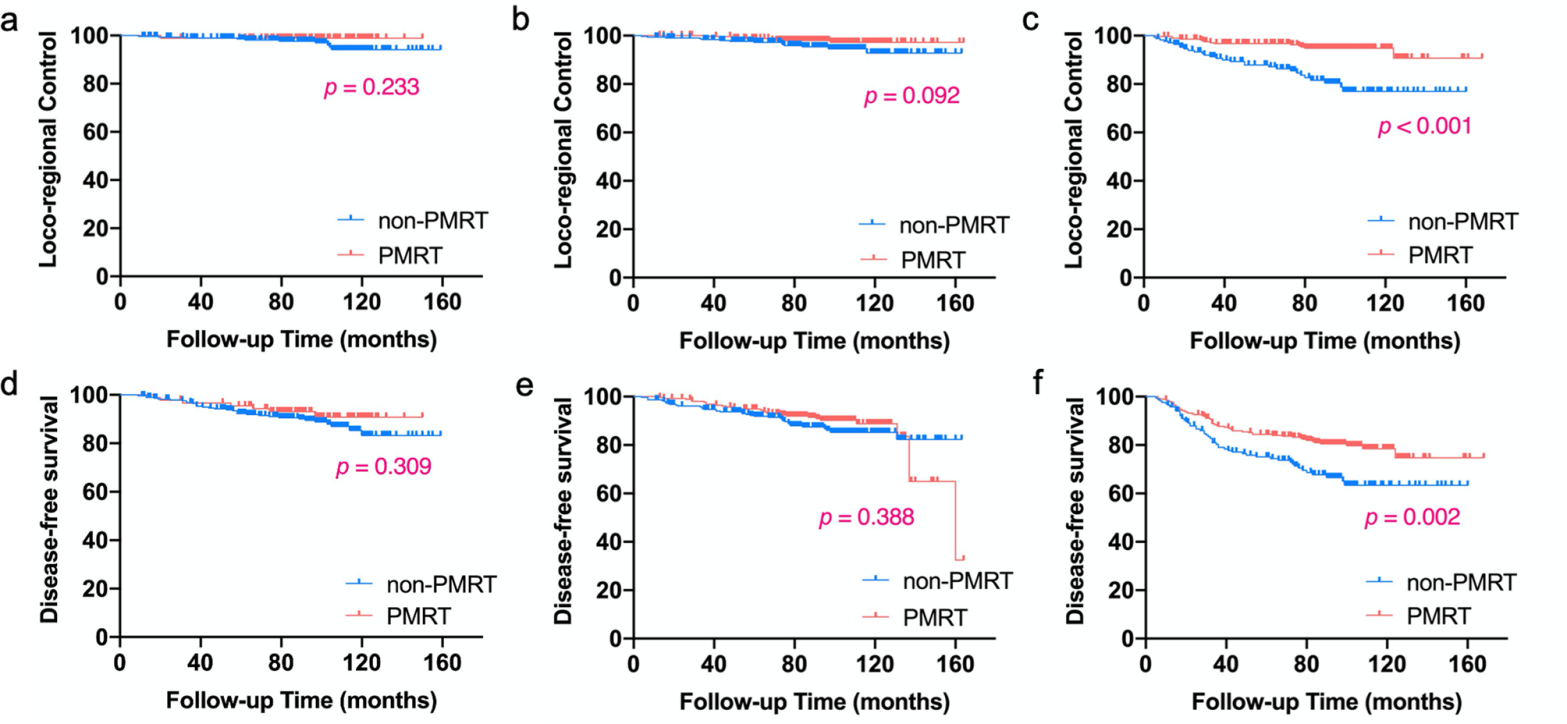
Kaplan–Meier analyses of locoregional control and disease-free survival stratified by recurrence risk. **a**, **d** low-risk group (no risk factor); **b**, **e** intermediate-risk group (1 risk factor); **c**, **f** high-risk group (2–4 risk factors). *PMRT* postmastectomy radiotherapy.

Figure 2 presents the Kaplan–Meier analyses of LRR and DFS stratified by recurrence risk. The 7.7-year LRC for patients in low-, intermediate-, and high-risk group was 97.7% / 98.9% (*p* = 0.233), 95.3% / 98.0% (*p* = 0.092), and 80.3% / 94.8% (*p* < 0.001) in the non-PMRT and PMRT subgroups, respectively. Meanwhile, PMRT was significantly associated with a longer DFS time in high-risk patients, with 7.7-year DFS of 66.6% in non-PMRT subgroup and 80.5% in PMRT subgroup (*p* = 0.002). However, no benefit from PMRT was observed in low-risk (*p* = 0.309) and intermediate-risk (*p* = 0.388) patients.

## Discussion

Our study analyzes the LRC and survival outcomes among 1474 breast cancer patients who underwent mastectomy and were pathologically staged T1-2N1, and makes PMRT recommendations according to risk stratification. The result demonstrated a 7.7-year LRC of 94.9% and DFS of 85.4% for the entire cohort in the era of modern therapy. Besides, PMRT was proved to be an independent predictor of LRC and DFS, despite that more patients in the PMRT subgroup had unfavorable disease features, such as larger tumor size, 2–3 positive ALNs, LVI + , and unfavorable molecular subtypes. However, the benefit of PMRT was not statistically significant for the 7.7-year DFS in the entire population (*p* = 0.335).

Evidence indicating that PMRT improves outcomes of patients with pT1-2N1 disease spans two treatment eras. The EBCTCG firstly powerfully proved the value of PMRT for node-positive patients in a meta-analysis including individual data of 8135 women from 22 trials during the period of 1964–86. The subgroup analysis of 1314 pN1 patients substantiated a reduction of 10-year LRR from 20.3 to 3.8%, 10-year overall recurrence from 45.7 to 34.2%, and 20-year breast cancer mortality from 50.2 to 42.3% with the application of PMRT [2]. Advanced analysis of EBCTCG indicated that PMRT could improve survival of node-positive patients regardless of the number of affected nodes and administration of adjuvant chemotherapy [12]. Another proof is the subgroup analysis of DBCG 82 b & c trial conducted between 1982 and 90. In the analysis of 1152 pN1 patients, PMRT reduced the 15-year locoregional failure (LRF) rate from 27 to 4% and improved the 15-year survival from 48 to 57% [13]. However, for patients since 1996, lower LRF rates were observed due to newer systemic treatment, modern RT techniques, and meticulous attention to surgical margins. In accordance with our data, recent studies reported that the estimated 5- and 10-year LRCs were 91–97% and 89–95% respectively in the absence of PMRT [14–20], which have an improvement compared with historic studies mentioned above. Therefore, several prospective and retrospective studies explored the value of PMRT in the era of modern therapy. The recently published EORTC 22922/10925 trial, which included 4004 patients, with 23.9% receiving mastectomy and 43.1% having 1–3 positive lymph nodes, revealed that the addition of regional nodes irradiation significantly reduced 15-year recurrence and breast cancer mortality [1]. Besides, in the subgroup analysis of BCIRG-005, PMRT improved 10-year LRC from 91 to 98% (*p* = 0.001) in pT1-2N1 patients [19]. Luo C et al. reported that PMRT was significantly associated with decreased LRR (5-year cumulative incidence 1.6% vs. 6.0%; HR, 0.248; 95% CI, 0.121–0.509; *p* < 0.001) in early staged patients who underwent mastectomy [21]. However, with the increased uptake of preoperative systemic therapy (PST), it has posed new challenge for patient selection for PMRT in the modern era. To date, no Grade 1 evidence was available that allow PMRT to be omitted in T1-2N1 patients who have undergone PST. The subgroup analysis of NSABP B-18/B-27 revealed that the 10-year incidence of LRR was greater than 10% (10.6—14.7%) for mastectomy patients with cN1 who received PST, suggesting that perhaps PMRT should be routinely recommended in the subgroup of patients [22]. So together with our findings, these results confirmed the value of locoregional irradiation among pT1-2N1 patients in the modern treatment era.

Given such favorable LRC even in non-PMRT patients with pT1-2N1 breast cancer, a key clinical challenge is to determine whom can omit PMRT. In this study, patients were stratified by the following risk factors, including young age, tumor size of 3-5 cm, 2–3 positive ALNs, and high histological grade, which were recognized in the univariate and multivariate analysis. For high-risk patients (≥ 2 risk factors), PMRT significantly improved both LRC (*p* < 0.001) and DFS (*p* = 0.002). In the intermediate-risk group (1 risk factor), a trend toward improvement of LRC (*p* = 0.092) but not DFS (*p* = 0.388) was observed with the addition of PMRT. However, no benefit from PMRT was observed in low-risk patients (0 risk factor), with 7.7-year LRC of 98.9% / 97.7% (*p* = 0.233) and DFS of 93.0% / 89.5% (*p* = 0.309) in the PMRT and non-PMRT patients, respectively. The outcomes of our study were consistent with previous studies with similar designs. Park HJ et al. conducted a multicenter analysis of 1382 patients staged pT1-2N1 in Korea (KROG 14–23), and identified that age < 35 years, T2 stage, high tumor grade, close resection margin, triple-negative biological subtype, and 2–3 positive nodes were independent risk factors of LRR. Further analysis revealed that patients with 0–1, 2–3, and 4–6 risk factors owned the 5-year LRR of 3.6%, 7.5%, and 12.7%, respectively, and demonstrated that patients with 2 or more risk factors might benefit from PMRT [9]. Luo CX et al. included tumor size, number of positive nodes, ER status, histologic grade, and LVI status in a nomogram for predicting LRR in a cohort of 1141 cases, and found that PMRT was significantly correlated with decreased LRR only in the high-risk group (5-year LRR 2.2% vs. 14.9%, *p* < 0.001); the 5-year LRR was relatively low in the low-risk (0.4%) and intermediate-risk (6.1%) non-PMRT group, and no survival advantage was observed in these two risk groups [21].

Besides, the role of IMN irradiation (IMNI) is debated as well. The Danish Breast Cancer Cooperative Group (DBCG), which included patients with right-sided disease allocated to IMNI and left-sided disease allocated to no IMNI, demonstrated that the addition of IMNI could significantly improve 8-year overall survival in node-positive patients (75.9% vs 72.2%, *p* = 0.005). Advanced analysis showed that the effect of IMNI was more pronounced in patients with high risk of IMN metastasis [23]. In this study, we investigated the value of IMNI in 259 high-risk patients who received CW and regional nodes irradiation. Results showed that no better outcomes of LRC (*p* = 0.759) or DFS (*p* = 0.816) were observed with the additional direction of IMNI. Therefore, for early staged patients with 1–3 positive nodes, randomized studies are wanted to assess the value of IMNI.

Unfavorable molecular subtypes including triple-negative and HER2-positive breast cancer are considered to be predictors of poor outcomes in many studies. Our analysis failed to recognize these biologic subtypes to be statistically significant predictors of poor LRC and DFS. The possible reason might be the improvement of patients’ survival with the application of novel systemic therapy; in our study, almost all patients received 4–8 cycles of chemotherapy in triple-negative (97.4%; 154/158) and HER2-positive (99.2%; 121/122) subtypes. Unfortunately, only 47.4% (144/304) of HER2-overexpressed patients received anti-HER2 therapy for economic reasons. The subgroup analysis revealed that the use of anti-HER2 drugs significantly improved both LRC (*p* = 0.022) and DFS time (p < 0.001), as reported in other studies [24, 25]. In this study, no more than 5% of HR-positive patients did not receive adjuvant endocrine therapy because of PR positive only or ER weakly positive that didn’t meet the positive criteria eight years ago. In the HR-positive subtype, we confirmed that the application of endocrine drugs was associated with a longer DFS interval (*p* = 0.015), but not a better LRC (*p* = 0.121).

Our study still has certain limitations, including its retrospective nature, limited follow-up time, and loss of individual data. To reduce the deviation of data missing, we conducted the univariate analysis prior to the multivariate analysis and thus maximally reduced data deletion in the calculating process. Moreover, we performed sensitivity analysis using the EM algorithm and yielded a complete dataset, the reanalysis results demonstrated that 1) younger age of ≤ 40 years (HR, 1.99; 95% CI, 1.18–3.36; *p* = 0.010), tumor size of 3–5 cm (HR, 1.70; 95% CI, 1.01–2.86; *p* = 0.048), histological grade 3 (HR, 1.39; 95% CI, 1.11–1.74; *p* = 0.004), 2–3 positive ALNs (HR, 2.45; 95% CI, 1.53–3.94; *p* < 0.001), and no PMRT delivery (HR, 3.12; 95% CI, 1.88–6.19; *p *< 0.001) remained the predictors of a poor LRC; 2) for patients with 0 (*p* = 0.238) or 1 (*p* = 0.173) risk factor, no benefit of PMRT was observed in improving LRC. Besides, due to the retrospective nature of this study, competing risk and imbalance between PMRT and non-PMRT groups need to be taken into consideration that might affect the results. When conducting competing risk analysis in consideration of death, the main competing event in the study, we demonstrated that the benefit from PMRT was retained in improving LRC (*p* = 0.003). Patients with unfavorable disease features were more likely to be directed to PMRT as mentioned above. This study stratified patients with numbers of risk factors they had, the selection bias was partially overcome in low- and intermediate-risk patients who owned 0 or 1 risk factor. In high-risk group, although patients in PMRT subgroup had more risk factors than non-PMRT subgroup, PMRT significantly improved LRC, which potently confirmed the value of PMRT. Despite such limitations, our study offers LRC and DFS estimates and makes PMRT recommendations according to risk stratification in the era of modern medicine. The retrospective-prospective study of NSABP B-14 and B-20 revealed that recurrence score (RS) of 21-gene OncotypeDX was efficient in predicting LRR, which leads to an era of precision medicine [26]. Ongoing trials in patients with T1-2 breast cancer and 1 to 3 positive nodes are expected to provide us more evidence of adjuvant RT. Of these, CCTG MA39 (TAILOR RT) trial will help to clarify the feasibility of omitting RT in low-risk patients with the assessment of RS. The current study discussed the role of PMRT without PST, however, another topic, the value of PMRT in patients submitted to PST, will be clarified in the ongoing NSABP B-51 (RTOG 1304) study, which included patients with cN1 disease pretreatment and achieving ypN0 after PST.

## Conclusions

This study demonstrates favorable LRC and DFS in patients who underwent mastectomy and were staged pT1-2N1 in the era of modern systemic therapy. On multivariate analysis, factors associated with increased recurrence risk include young age, tumor size of 3–5 cm, histological grade 3, and 2–3 positive lymph nodes. Stratifying by these factors, the risk group was significantly associated with LRR risk and RT benefit. Low- to intermediate-risk patients had a small benefit from PMRT and might consider omitting PMRT; High-risk patients had a greater benefit and therefore should consider the routine use of PMRT. Further prospective investigations are needed to improve risk stratification and estimates of RT benefit in individuals with pT1-2N1 breast cancer after mastectomy.

## Data Availability

The datasets generated and/or analyzed during the current study are available from the corresponding author on reasonable request.

## Abbreviations

PMRT: Postmastectomy radiotherapy
ALNs: Axillary lymph nodes
EBCTCG: The Early Breast Cancer Trialists’ Collaborative Group
LRR: Locoregional recurrence
LVI: Lymph-vascular invasion
ER: Estrogen receptor
PR: Progesterone receptor
HER2: Human epidermal receptor 2
IHC: Immunohistochemistry
CW: Chest wall
SCV: Supraclavicular
ICV: Infraclavicular
IMN: Internal mammary nodes
LRC: Locoregional control
DM: Distant metastases
DFS: Disease-free survival
RT: Radiotherapy
LRF: Locoregional failure
RS: Recurrence score
PST: Preoperative systemic therapy
IMNI: IMN irradiation
